# Memory Dynamics in Attractor Networks

**DOI:** 10.1155/2015/191745

**Published:** 2015-04-19

**Authors:** Guoqi Li, Kiruthika Ramanathan, Ning Ning, Luping Shi, Changyun Wen

**Affiliations:** ^1^Centre for Brain Inspired Computing Research (CBICR), Department of Precision Instrument, Tsinghua University, Beijing 100084, China; ^2^Department of Advanced Concepts and Nanotechnology (ACN), Data Storage Institute, A∗STAR, 5 Engineer Drive 1, Singapore 117608; ^3^School of Electrical and Electronic Engineering, Nanyang Technological University, Singapore 639798

## Abstract

As can be represented by neurons and their synaptic connections, attractor networks are widely believed to underlie biological memory systems and have been used extensively in recent years to model the storage and retrieval process of memory. In this paper, we propose a new energy function, which is nonnegative and attains zero values only at the desired memory patterns. An attractor network is designed based on the proposed energy function. It is shown that the desired memory patterns are stored as the stable equilibrium points of the attractor network. To retrieve a memory pattern, an initial stimulus input is presented to the network, and its states converge to one of stable equilibrium points. Consequently, the existence of the spurious points, that is, local maxima, saddle points, or other local minima which are undesired memory patterns, can be avoided. The simulation results show the effectiveness of the proposed method.

## 1. Introduction

Memory is a fundamental component of our human brain; how to simulate the human memory process has attracted many scientists attention in the research of cognitive systems and architectures [1, 2]. Attractor networks [3–6] have been one of the most popular models for memory storage and retrieval in recent decades since the hypothesis of attractor dynamics is supported and observed in the neocortex and hippocanpus in various memory experiments [6–9]. In general, an attractor network is a network of recurrently connected nodes in a biological network, whose states may settle to some stable patterns. One distinguish advantage is that the network can be represented by neurons and their synaptic connections. The particular pattern of such a recurrent network, which is called its “attractor” [10–13], can be stationary, time varying, or even stochastic. In theoretical neuroscience, different kinds of attractor neural networks have been associated with different functions, such as memory, motor behavior, and classification. In this paper, we consider the patterns or the so-called attractors as the stationary memory patterns stored in the dynamic system, which allows us to employ methods in dynamical systems to quantitatively analyze the characteristics such as the stability and robustness of the network.

Usually researchers design an attractor network and then propose its energy function, often called Lyapnov function, to analyze the network [14–16], since the energy function plays a very important role in analyzing the network stability and robustness. In this paper, we design the memory dynamics in an opposite direction. We first propose an energy function and then design the attractor network. The energy function actually contains the desired memory patterns we wish to store. Different from Hopfield networks [10, 14], we introduce multiplicative algebra into the attractor network. This is biologically possible since both addition algebra and multiplication algebra are the simplest and the most widespread of all operations in the nervous system [17]. In addition, the value of our proposed energy function is nonnegative and attains zero values only at the patterns stored in the network. This makes it easy to distinguish the desired memory patterns from some other possible undesired patterns which are called spurious points [18]. It is shown that the memory patterns are stored as the stable equilibrium points of the dynamical attractor networks, which are also the local minimum points of the energy function. Compared with existing results in attractor networks [7, 10, 14], the patterns are not necessarily binary and uncorrelated in this paper. Binary patterns simplify the network design significantly as seen in [14]. Also, the uncorrelated patterns give a minimum interactions of the interactions of the network, which makes its behavior analysis much easier as seen in [7, 19].

On the other hand, when a stimulus pattern is presented as an initial input of the dynamic system, the states of the system will converge to a particular stored attractor iteratively. This process is called associative memory retrieval. The “associative” means that the memory we retrieved was by its informational content rather than by names, addresses, or relative positions. One very touchy problem in the retrieval process is how to overcome the problem that the system states may converge to spurious points; see, for example, [18, 20]. Usually, there are two kinds of equilibrium points for a dynamic system, the stable ones and unstable ones. It is shown that only the stable equilibrium points, that is, the local minimum points, of the energy function exist in the proposed designed system. We also prove that those local minima are only the memory patterns stored in the network. Thus, the spurious points, that is, local maxima, saddle points, or other local minima which are the undesired memory patterns, can be avoided.

The contributions of this paper are summarized as follows. Firstly, we have proposed a new energy function different from the energy function in Hopfield networks. This makes it easy to differentiate memory patterns from possible spurious points. Secondly, we have presented an attractor network design based on the proposed energy function. The patterns stored in the attractor network can be nonbinary and either correlated or uncorrelated. Finally, we have proven that, when an arbitrary input stimulus is presented to the designed attractor network, the states converge to one of the stored patterns. This implies that there are no spurious points in the designed dynamical systems.

The rest of the paper is organized as follows. Some background knowledge is reviewed in Section 2.  Section 3 introduces the main design method of the proposed attractor network. The convergence properties are analyzed in Section 4. The simulation examples are shown in Section 5. Finally, the paper is concluded in Section 6.

## 2. Background Knowledge

### 2.1. Multiplication Algebra in Nervous Systems

Addition algebra is both the simplest and one of the most widespread of all operations in nervous systems. However, as pointed in [17], a number of biological mechanisms could, in theory, implement a multiplication algebra. Actually multiplication can be implemented based on addition. For example, when we multiply two signals *x* and *y*, we can logarithmically transform the two, add the result,and then apply an exponential:

(1) $$\begin{array}{c} xy=\exp^{\left(\log\left(x\right)+\log\left(y\right)\right)}. \end{array}$$

Thus, later it can be seen that the memory dynamics in our proposed attractor network could be implemented in a network with neurons and synapses.

### 2.2. Notations and Definitions

Denote a nonlinear function *f*(**x**) and a pattern **x** = [*x*_1_ ⋯ *x*_*m*_]^*T*^ ∈ *R* ^*m*^ where *m* is the dimension. For a nonlinear dynamical system $\dot{x}=-f(x)$, we have the following definitions.

**Definition 1 .**

A pattern **x** ^0^ ∈ *R* ^*m*^ is called an equilibrium point of $\dot{x}=-f(x)$, if *f*(**x**) is a zero vector at **x** = **x** ^0^, which is denoted as *f*(**x** ^0^) = 0.

**Definition 2 .**

A pattern **x** ^0^ ∈ *R* ^*m*^ is called a stable equilibrium point of $\dot{x}=-f(x)$, if *f*(**x**) = 0 at **x** ^0^ and the Jacobian matrix at **x** ^0^ is a positive definite matrix.

**Definition 3 .**

A pattern **x** ^0^ ∈ *R* ^*m*^ is called an unstable equilibrium point (saddle point) of $\dot{x}=-f(x)$, if *f*(**x**) = 0 at **x** ^0^ while the Jacobian matrix at **x** ^0^ is a negative semidefinite or an indefinite matrix.

## 3. A New Energy Function and the Attractor Network

A classical energy based attractor network is the Hopfield network invented by Hopfield [10, 14], which serves as content addressable memory systems with binary threshold nodes. Although Hopfield networks are guaranteed to converge to a local minimum, they may converge to a spurious point (undesired memory pattern) rather than a stored pattern (desired memory pattern). To solve this problem, we propose a new energy function, and an attractor network is then designed based on the proposed energy function, in which the patterns are not necessarily binary and uncorrelated. Finally it is concluded that the memory patterns are the stable equilibrium points of the dynamic system and spurious points can be avoided.

Assume that **x** ^1^,…, **x** ^*k*^,…, **x** ^*n*^ with **x** ^*k*^ = [*x*_1_^*k*^ ⋯ *x*_*m*_^*k*^]^*T*^ ∈ *R* ^*m*^ are *n* different stationary patterns that we wish to store. Our objective is to design an attractor network to store these patterns such that **x** ^*k*^ for *k* = 1,…, *n* can be retrieved when an input stimulus is located around the neighborhood of **x** ^*k*^. Before presenting our proposed method, we would like to point out that an ideal attractor network should preserve the following two properties.

**Property 1 .**

**x** ^1^,…, **x** ^*k*^,…, **x** ^*n*^ are stable equilibrium points of the attractor network.

**Property 2 .**

**x** ^1^,…, **x** ^*k*^,…, **x** ^*n*^ are the only stable equilibrium points of the attractor network.

The energy function is designed as the following form:

(2) $$\begin{array}{c} F\left(\;x\;\right)=\overset{k=n}{\underset{k=1}{\prod }}d_{k}\left(\;x\;\right)=\overset{k=n}{\underset{k=1}{\prod }}\left[\;{\left(\;x-x^{k}\;\right)}^{T}\left(\;x-x^{k}\;\right)\;\right], \end{array}$$

where *d*_*k*_(**x**) = (**x** − **x** ^*k*^)^*T*^(**x** − **x** ^*k*^) is an Euclidian squared distance between **x** and **x** ^*k*^. The energy function is the product of the distance *d*_*k*_(**x**) for *k* = 1,…, *n*. For the energy function *F*(**x**), it can be checked that ∀**x** ∈ *R* ^*m*^, *F*(**x**) > 0, and *F*(**x**) = 0 if and only if **x** = **x** ^*k*^ for *k* = 1,…, *n*.

**Remark 4 .**

As mentioned in the Introduction, compared with the energy function in the Hopfield network [10, 14], the value of the proposed energy function *F*(**x**) is nonnegative and attains zero value only on the memory patterns stored in the network. This makes it easy to distinguish the memory patterns from spurious points [18] which may exist in an attractor network.

Note that the energy function attains its minimum only on the memory patterns **x** ^1^,…, **x** ^*n*^. This inspires us to design a dynamical system which can make the energy function decrease iteratively. The gradient ∇*F*(**x**) and the Hessian matrix ∇^2^ *F*(**x**) are given by

(3) ∇Fx=2∑j=1j=n∏k=1,k≠jk=ndkxx−xj∇2Fx=2∑j=1j=n∏k=1,k≠ji=ndkx·I+4∑j=1j=n∑k′=1,k′≠jk′=n∏k=1,k≠j,k≠k′i=ndkx·x−xk′x−xjT,

where *I* is a *m* dimensional identity matrix. Let **v** = [*v*_1_ ⋯ *v*_*m*_]^*T*^ be a random noise vector such that

(4) $$\begin{array}{c} \max\left(\;\left|\;v\;\right|\;\right)=\max\left(\begin{array}{ccc} \left|v_{1}\right| & \cdots & \left|v_{m}\right| \end{array}\right\}<v_{\max}, \end{array}$$

where **v**_max⁡_ is a chosen small positive constant. Now an attractor network that decreases the energy function *F*(**x**) iteratively is represented as

(5) $$\begin{array}{c} \dot{x}=-\nabla F\left(\;x\;\right)+\kappa \cdot F\left(\;x\;\right)\cdot v, \end{array}$$

where the scalar constant *κ* is chosen as

(6) $$\begin{array}{c} \kappa =\left\{\begin{array}{cc} 0 & \text{if}\;\;\max\left(\left|\nabla F\left(x\right)\right|\right)>0\\ 1 & \text{if}\;\;\max\left(\left|\nabla F\left(x\right)\right|\right)\leq 0. \end{array}\right. \end{array}$$

We rewrote the differential equation for each neuron *x*_*i*_ as

(7) $$\begin{array}{c} {\dot{x}}_{i}=-\overset{n}{\underset{j=1}{\sum }}\omega_{ij}\left(\;x_{i}-x_{i}^{j}\;\right)+\kappa \cdot F\left(\;x\;\right)\cdot v_{i} \end{array}$$

for *i* = 1,…, *m* with

(8) $$\begin{array}{c} \omega_{ij}=2\left[\;\overset{k=n}{\underset{k=1,k\neq j}{\prod }}d_{k}\left(\;x\;\right)\;\right]. \end{array}$$

Equations (7) and (8) imply that *i*th neuron updates its state *x*_*i*_ according to the synaptic inputs collected from other neurons via synaptic connection strength *ω*_*ij*_, in the presence of a random noise *v*_*i*_.

**Remark 5 .**

The Hopfield model has the advantage that it can be represented by neurons and their synaptic connections. Through carefully observing (5)–(8), we note that our proposed attractor network can also be represented by neurons and their synaptic connections by introducing the multiplication algebra in Section 2.1. In addition, the value of our proposed energy function is nonnegative and attains zero values only at the patterns stored in the network. This makes it easy to distinguish the memory patterns from some other possible undesired patterns which are called spurious points [18]. It is shown that the memory patterns are stored as the stable equilibrium points of the dynamical system, which are also the local minimum points of the energy function. Compared with existing results in attractor networks [7, 10, 14], the patterns are not necessarily binary and uncorrelated in this paper. Binary patterns simplify the network design significantly as seen in [14]. Also, the uncorrelated patterns give a minimum interaction of the interactions of the network, which makes its behavior analysis much easier as seen in [7, 19].

**Remark 6 .**

As mentioned earlier, there are two kinds of equilibrium points for a dynamic system: stable ones and unstable ones. In the next section, it will be shown that only the stable equilibrium points exist in the above dynamical system. For an arbitrary initial stimulus, the states of the dynamical system converge to one of its stable equilibrium points, that is, the local minimum points of the energy function, which cannot be its local maximum points or saddle points of the energy function. Thus, Property 1 can be achieved in the design. In the next section, it will also be proven that Property 2 can also be achieved in the design; that is, **x** ^1^,…, **x** ^*n*^ are the only local minimum points of *F*(**x**).

## 4. Convergence Analysis

**Lemma 7 .**

For square matrix *A* ∈ *R* ^*m*×*m*^, assume that the eigenvalues of *A* are not all the same. If tr⁡(*A*) ≤ 0, *A* cannot be a positive semidefinite or positive definite matrix.

**Proof**

Denote the eigenvalues of *A* as *λ*_1_,…, *λ*_*m*_. Note that tr⁡(*A*) = ∑_*i*=1_^*i*=*m*^ *λ*_*i*_. If tr⁡(*A*) = ∑_*i*=1_^*i*=*m*^ *λ*_*i*_ < 0, then ∃*λ*_*i*_ such that *λ*_*i*_ < 0. If tr⁡(*A*) = ∑_*i*=1_^*i*=*m*^ *λ*_*i*_ = 0, there also exists ∃*λ*_*i*_ such that *λ*_*i*_ < 0 since *λ*_1_,…, *λ*_*m*_ cannot be identically equal. Then this lemma holds.

**Lemma 8 .**

For conformable matrices *A*, *B*, and *C*, tr⁡(*ABC*) = tr⁡(*BCA*) = tr⁡(*CAB*). Also, if matrices *A* and *B* are addable, tr⁡(*A* + *B*) = tr⁡(*A*) + tr⁡(*B*) [21].

**Proof**

To prove tr⁡(*ABC*) = tr⁡(*BCA*) = tr⁡(*CAB*), actually we only need to prove that tr⁡(*AB*) = tr⁡(*BA*). As tr⁡(*AB*) = Σ_*i*_Σ_*i*_ *A*_*ij*_ *B*_*ji*_ = tr⁡(*BA*) and tr⁡(*A* + *B*) = Σ_*i*_(*A*_*ii*_ + *B*_*ii*_) = Σ_*i*_(*A*_*ii*_) + Σ_*i*_(*B*_*ii*_) = tr⁡(*A*) + tr⁡(*B*), this lemma holds.

**Theorem 9 .**

The states of the attractor network in (5)–(8) converge to an stable equilibrium point **x** ^∗^ ∈ {**x** ^1^,…, **x** ^*m*^} such that ∇*F*(**x** ^∗^) = 0, *F*(**x** ^∗^) = 0, and ∇^2^ *F*(**x** ^∗^) is positive definite which is denoted as ∇^2^ *F*(**x** ^∗^) > 0.

**Proof**

By Definition 1, the equilibrium points of system (7) are such that

(9) $$\begin{array}{c} -\nabla F\left(\;x\;\right)+\kappa \cdot F\left(\;x\;\right)\cdot v=0. \end{array}$$

As **v** is a random vector and *κ* is chosen by (6), a point is an equilibrium point of system (7) if and only if

(10) ∇Fx0,Fx=0.

*F*(**x**) = 0 gives us that all the possible equilibrium points of (7) are those points at which the values of energy function *F*(**x**) are zero; that is, **x** ^∗^ ∈ {**x** ^1^,…, **x** ^*m*^}. In addition, the Jacobian matrix at a point **x** ^*i*^ is the Hessian matrix ∇^2^ *F*(**x** ^*i*^). Then, it is easy to obtain that

(11) ∇Fxi0,∇2Fxi2∏k=1,k≠ik=ndkxi·I>0.

So **x** ^∗^ is a stable equilibrium point of the attractor network in (5) to (8) based on Definition 2.

**Theorem 10 .**

For the case that the dimension *m* ≤ 2, **x**_1_,…, **x**_*n*_ are the only local minimum points of *F*(**x**).

**Proof**

Obviously, **x**_1_,…, **x**_*n*_ are local minimum points (and also global minimum points) of *F*(**x**). We now prove that *F*(**x**) has no other local minimum points. Let

(12) A=2∑j=1j=n∏k=1,k≠jk=ndkx·I,B=4∑j=1j=n∑k′=1,k′≠jk′=n·∏k=1,k≠j,k≠k′k=ndkx·x−xk′x−xjT.

It can be obtained that ∇^2^ *F*(**x**) = *A* + *B*. It is also known that the dimension of diagonal matrix *A* is *m* × *m*; then

(13) $$\begin{array}{c} \mathrm{tr}\left(\;A\;\right)=m\cdot 2\overset{j=n}{\underset{j=1}{\sum }}\left[\;\overset{k=n}{\underset{k=1,k\neq j}{\prod }}d_{k}\left(\;x\;\right)\;\right]. \end{array}$$

Also, Lemma 8 gives that

(14) tr⁡B=4∑j=1j=n∑k′=1,k′≠jk′=n∏k=1,k≠j,k≠k′k=ndkx·x−xk′Tx−xj.

Now we assume that there is a point which is different from **x**_1_,…, **x**_*n*_ but satisfies that ∇*F*(**x** ^∗^) = 0. This implies that (∇*F*(**x** ^∗^))^*T*^∇*F*(**x** ^∗^) = 0. This is to say that

(15) $$\begin{array}{c} G\left(\;x^{*}\;\right)\cdot \overset{k=n}{\underset{k=1}{\prod }}{\left(\;x-x^{k}\;\right)}^{T}\left(\;x-x^{k}\;\right)=0, \end{array}$$

where

(16) GX∗=4∑j=1j=n∏k=1,k≠jk=ndkx+4∑j=1j=n∑k′=1,k′≠jk′=n∏k=1,k≠j,k≠k′i=ndkx∗·x∗−xk′Tx∗−xj.

Thus, we have *G*(**x** ^∗^) = 0 if **x** ^∗^ is different from **x** ^1^,…, **x** ^*n*^. Let *A* ^∗^ and *B* ^∗^ be *A* and *B* at **x** = **x** ^∗^. Combining (13)-(14) and (16), it can be obtained that

(17) $$\begin{array}{c} G\left(\;x^{*}\;\right)=\mathrm{tr}\left(\;\frac{2}{m}\cdot A^{*}+B^{*}\;\right)=0. \end{array}$$

Since **x** ^1^,…, **x** ^*n*^ are all different from each other, the eigenvalues of ∇^2^ *F*(**x** ^∗^) cannot be all the same. When *m* = 1, we have *G*(**x** ^∗^) = tr⁡(2*A* ^∗^ + *B* ^∗^) = 0, which implies tr⁡(*A* ^∗^ + *B* ^∗^) < 0 since *A* ^∗^ is a positive definite matrix. Similarly, we have tr⁡(∇^2^ *F*(**x** ^∗^)) = 0 when *m* = 2. From Lemma 7, matrix ∇^2^ *F*(**x** ^∗^) cannot be a positive definite matrix. It is a seminegative definite or an indefinite matrix. This implies that **x** ^∗^ cannot be a local minimum point though ∇*F*(**x** ^∗^) = 0. It can be a local maximum point or a saddle point of *F*(**x**).

**Remark 11 .**

If the dimension *m* > 2, tr⁡((2/*m*) · *A* ^∗^ + *B* ^∗^) = 0 does not directly imply that ∇^2^ *F*(**x** ^∗^) is a negative semidefinite or an indefinite matrix. Let *P*_*f*_ ∈ *R* ^2×*n*^ be a full-row rank projection matrix, which projects a vector into a two-dimensional plane. The following theorem shows that **x** ^1^,…, **x** ^*n*^ are still the only local minimum points of *F*(**x**).

**Theorem 12 .**

For the case that *m* > 2, **x** ^1^,…, **x** ^*n*^ are also the only local minimum points of *F*(**x**).

**Proof**

Let $\tilde{x}=P_{f}x$ and ${\tilde{x}}^{1},\ldots ,{\tilde{x}}^{n}$ be the corresponding projection of the local minimum points **x** ^1^,…, **x** ^*n*^ in the two-dimensional plane. Then, *F*(**x**) and ∇*F*(**x**) become $F(\tilde{x})$ and $\nabla F(\tilde{x})$, respectively, on this two-dimensional plane. It can be obtained that

(18) ∇Fx~Pf∇Fx∇2Fx~=Pf∇2FxPfT.

If ∇*F*(**x**) = 0 and ∇^2^ *F*(**x**) > 0, then $\nabla F(\tilde{x})=0$ and $\nabla^{2}F(\tilde{x})>0$, which means that the local minimum points of *F*(**x**) in a higher dimensional space must be also the local minimum points of $F(\tilde{X})$ in the two-dimensional plane while the converse is not true. Obviously, **x** ^1^,…, **x** ^*n*^ are the local minima of $F(\tilde{x})$.

Assume that there is a point **x** ^∗^ which is different from **x** ^1^,…, **x** ^*n*^ but a local minimum of *F*(**x**). There exists a full-row rank projection matrix *P*_*f*_^∗^ ∈ *R* ^2×*n*^ such that ${\tilde{x}}^{*}=P_{f}^{*}x^{*}$. The full-row rank of *P*_*f*_^∗^ implies that ${\tilde{x}}^{*}$ is different from ${\tilde{x}}^{1},\ldots ,{\tilde{x}}^{i},\ldots ,{\tilde{x}}^{n}$, where ${\tilde{x}}_{i}=P_{f}^{*}x_{i}$ for *i* = 1,…, *n* are the local minimum points of $F(\tilde{x})=F(P_{f}^{*}x)$. However, this is impossible by Theorem 10. Thus, there is no other local minimum point of *F*(**x**). If a point **x** ^∗^ satisfies that ∇*F*(**x** ^∗^) = 0 but it is different from **x** ^1^,…, **x** ^*n*^, it can be only a local maximum point or a saddle point. So this theorem holds.

## 5. Simulation Results

**Example 1 .**

Design a nonlinear dynamical system whose attractors are **x** ^1^,…, **x** ^*n*^ ∈ *R* ^*m*^ with *n* = 2, *m* = 1.

This corresponds to the one-dimensional case. The energy function is constructed as *F*(**x**) = (**x** − **x** ^1^)^2^(**x** − **x** ^2^)^2^ from (2) with ∇*F*(*X*) and ∇^2^ *F*(*X*) being given by

(19) ∇Fx=2x−x12x−x2+2x−x22x−x1,∇2Fx=2x−x12+2x−x22+4x−x2x−x1+4x−x1x−x2,

respectively. The dynamic system can be then designed in (5)–(8). By solving ∇*F*(**x** ^∗^) = 0, we have **x** ^∗^ = **x** ^1^, **x** ^∗^ = **x** ^2^, or **x** ^∗^ = (**x** ^1^ + **x** ^2^)/2 as analyzed in Section 3. From

(20) $$\begin{array}{c} {\left.\;\nabla^{2}F\left(\;x\;\right)\;\right|}_{x=x^{*}=\left(\;x^{1}+x^{2}\;\right)/2}<0, \end{array}$$

**Figure 1:**
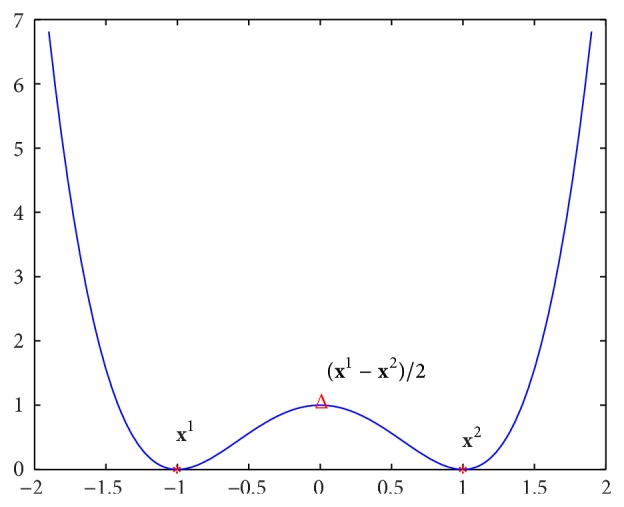
Energy function for one-dimensional case.

**x** ^∗^ = (**x** ^1^ + **x** ^2^)/2 is a local maximum point of *F*(**x**). So **x** ^1^ and **x** ^2^ are the only two local minimum points of *F*(**x**). The energy function is shown in Figure 1 where “∗” denotes the local minimum point and “Δ” denotes the local maximum or the saddle point. From Figure 1, when the initial stimulus **x** < (**x** ^1^ + **x** ^2^)/2, the states converge to **x** ^1^; when **x** > (**x** ^1^ + **x** ^2^)/2, the states converge to **x** ^2^. If the initial stimulus **x** = (**x** ^1^ + **x** ^2^)/2, the states can converge to either **x** ^1^ or **x** ^2^, which depends on the random noise *v*_1_ in (7).

**Example 2 .**

Example 1, analyze the dynamic system when *n* = 2, *m* = 2 and *n* = 2, *m* = 3, respectively.

**Case 1 .**

**x** ^1^ = [−0.5 −0.5]′ and **x** ^2^ = [0.5 0.5]′. Similar to Example 1, the energy function is constructed as *F*(**x**) = *d*_1_(**x**)*d*_2_(**x**) with ∇*F*(**x**) and ∇^2^ *F*(**x**) being given by

(21) ∇Fx=2d1xx−x2+2d2xx−x1,∇2Fx=2x−x1Tx−x1·I+2x−x2Tx−x2·I+4x−x2x−x1T+4x−x1x−x2T.

Solving ∇*F*(**x** ^∗^) = 0 gives **x** ^∗^ = **x** ^1^, **x** ^∗^ = **x** ^2^, or *X* ^∗^ = (**x** ^1^ + **x** ^2^)/2. When *X* ^∗^ = (**x** ^1^ + **x** ^2^)/2, we have

(22) $$\begin{array}{c} \nabla^{2}F\left(\;x^{*}\;\right)=\left[\begin{array}{cc} 0 & -2\\ -2 & 0 \end{array}\right]. \end{array}$$

**Figure 2:**
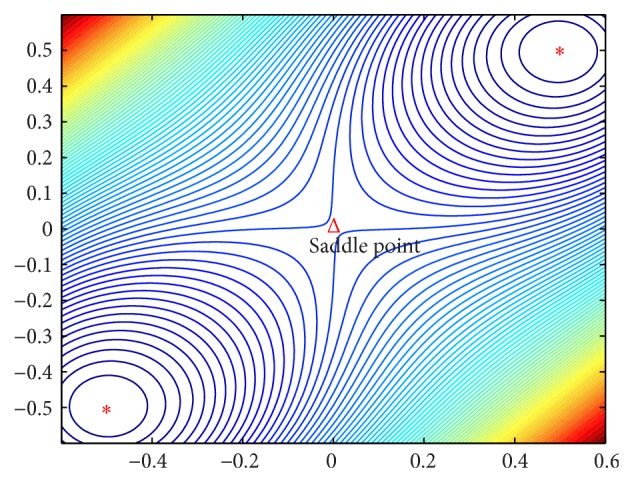
The contours of the energy function for Example 2.

The two eigenvalues of ∇^2^ *F*(**x** ^∗^) are *λ*_1_ = 2 and *λ*_2_ = −2. This is consistent with Theorem 10, which gives tr⁡(∇^2^ *F*(**x** ^∗^)) = 0.  Figure 2 shows the contour map of the energy function in a two-dimensional space. The contour lines around a saddle point look like a horse saddle. It can be concluded that **x** ^1^ and **x** ^2^ are two attractors while (**x** ^1^ + **x** ^2^)/2 is not an equilibrium point of system (5)–(8) by Theorem 9. The term *κ* · *F*(**x**)**v** guarantees that the attractor network (5)–(8) cannot stay at the point (**x** ^1^ + **x** ^2^)/2. But from Theorem 10, we know that (**x** ^1^ + **x** ^2^)/2 is a saddle point of *F*(**x**).

**Case 2 .**

**x** ^1^ = [−0.5 −0.5 −0.5]′ and **x** ^2^ = [0.5 0.5 0.5]′. In this case, *n* = 2, *m* = 3. When **x** ^∗^ = (**x** ^1^ + **x** ^2^)/2, we have

(23) $$\begin{array}{c} \nabla^{2}F\left(\;x^{*}\;\right)=\left[\begin{array}{ccc} 1 & -2 & -2\\ -2 & 1 & -2\\ -2 & -2 & 1 \end{array}\right]. \end{array}$$

Then **x** ^∗^ is still not an equilibrium point of system (5)–(8) but a saddle point of *F*(**x**) as the eigenvalue of ∇^2^ *F*(**x** ^∗^) is now *λ*_1_ = −3, *λ*_2_ = 3, and *λ*_3_ = 3. But in this case, we have tr⁡(∇^2^ *F*(**x** ^∗^)) > 0. So we cannot determine whether **x** ^∗^ is a saddle point or a local minimum point of *F*(**x**) only based on Theorem 10. However, we can determine that it is also a saddle point by Theorem 12. This is consistent with what we observed in simulation, as the eigenvalues of above ∇^2^ *F*(**x** ^∗^) is 3,3, −3, and thus **x** ^∗^ is a saddle point and cannot be a local minimum point of *F*(**x**).

**Example 3 .**

Design a nonlinear dynamic system whose attractors are **x** ^1^ = [2 0]′, $x^{2}={\left[-1\;\sqrt{3}\right]}^{\prime }$, and $x^{3}={\left[-\sqrt{3}\;-1\right]}^{\prime }\in R^{2}$.

We have *n* = 3 and *m* = 2 in this example. The energy function is constructed as *F*(**x**) = *d*_1_(**x**)*d*_2_(**x**)*d*_3_(**x**) with

(24) ∇Fx=2d1xd2xx−x3+2d1xd3xx−x2+2d2xd3xx−x1,∇2Fx=2d1xd2x·I+2d1xd3x·I+2d2xd3x·I+4x−x3x−x1Tx−x2Tx−x2+4x−x3x−x2Tx−x1Tx−x1+4x−x2x−x1Tx−x3Tx−x3+4x−x2x−x3Tx−x1Tx−x1+4x−x1x−x2Tx−x3Tx−x3+4x−x1x−x3Tx−x2Tx−x2

with its dynamical property described by the attractor network in (5)–(8). Theorem 9 tells us that **x** ^1^, **x** ^2^, and **x** ^3^ are the stable equilibrium points of *F*(**x**) and the attractor network in (5)–(8). However, *F*(**x**) has more saddle points but these saddle points are not equilibrium points (saddle points) of (5)–(8). To illustrate this, firstly, we find all the points such that ∇*F*(*X* ^∗^) = 0. If ∇*F*(*X* ^∗^) = 0, then (∇*F*(*X* ^∗^))^*T*^∇*F*(*X* ^∗^) = 0, which means

(25) $$\begin{array}{c} G\left(\;x^{*}\;\right)\cdot \overset{k=3}{\underset{k=1}{\prod }}d_{k}\left(\;x^{*}\;\right)=0, \end{array}$$

where

(26) Gx∗=2d1x∗d2x∗+2d1x∗d3x∗+2d2x∗d3x∗+4x∗−x3Tx∗−x1d2x∗+4x∗−x3TX∗−x2d1x∗+4x∗−x2Tx∗−x1d3x∗+4x∗−x2Tx∗−x3d1x∗+4x∗−x1Tx∗−x2d3x∗+4x∗−x1Tx∗−x3d2x∗.

We know that (∇*F*(**x** ^∗^))^*T*^∇*F*(**x** ^∗^) = 0 gives **x** ^∗^ = **x** ^1^, **x** ^∗^ = **x** ^2^, **x** ^∗^ = **x** ^3^ or *G*(**x** ^∗^) = 0. But *G*(**x** ^∗^) = 0 implies that

(27) $$\begin{array}{c} \mathrm{tr}\left(\;\nabla^{2}F\left(\;x^{*}\;\right)\;\right)=0. \end{array}$$

**Figure 3:**
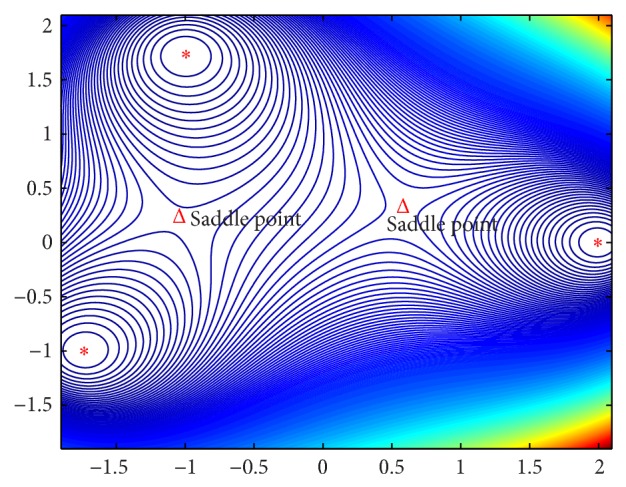
The contours of the energy function for Example 3.

Thus, **x** ^∗^ will be a saddle point of *F*(**x**) but not the attractor network in (5)–(8) if ∇*F*(**x** ^∗^) = 0 while **x** ^∗^ is different from **x** ^1^,…, **x** ^*n*^. As the attractor network in (5)–(8) does not have any saddle points from Theorem 9, usually, a saddle point of *F*(**x**) is located in between two local minimum points. As seen from Figure 3, two saddle points of *F*(**x**) are in between the three local minimum points on the plane. One is located about (−1.05,0.22) and the other is about (0.58,0.31).

## 6. Conclusion

The contributions of this paper are summarized as follows.

1. We have proposed a new energy function which includes the information of the stored patterns, and it is different from the energy function in Hopfield network. The proposed energy function makes it easy to differentiate memory patterns from possible spurious points.
2. We have presented an attractor network design based on the proposed energy function. The patterns stored in the attractor network can be nonbinary and either correlated or uncorrelated. The memory patterns are the attractors of the network and the equilibrium points of the dynamic system.
3. When an arbitrary input stimulus is presented to the designed attractor network, it has been proved that the states converge to one of the stored patterns. There are no spurious states in the designed dynamic systems.

Our future work is to construct a biological plausible dynamical system in hardware (neuromorphic chip) which can stimulate the behavior of the designed network. This sheds new lights on the research towards the realization of artificial cognitive memory.

## References

[1] Shi L. P., Yi K. J., Ramanathan K. (2011). Artificial cognitive memory-changing from density driven to functionality driven. *Applied Physics A: Materials Science and Processing*.

[2] Li G., Ning N., Ramanathan K., He W., Pan L., Shi L. (2013). Behind the magical numbers: hierarchical chunking and the human working memory capacity. *International Journal of Neural Systems*.

[3] Amit D. J., Gutfreund H., Sompolinsky H. (1985). Spin-glass models of neural networks. *Physical Review, A: Third Series*.

[4] Amit D. J., Mongillo G. (2003). Spike-driven synaptic dynamics generating working memory states. *Neural Computation*.

[5] Poucet B., Save E. (2005). Attractors in memory. *Science*.

[6] Tsodyks M. (2005). Attractor neural networks and spatial maps in hippocampus. *Neuron*.

[7] Tang H., Li H., Yan R. (2010). Memory dynamics in attractor networks with saliency weights. *Neural Computation*.

[8] Miyashita Y. (1988). Neuronal correlate of visual associative long-term memory in the primate temporal cortex. *Nature*.

[9] Bakker A., Kirwan C. B., Miller M., Stark C. E. L. (1999). Pattern separation in the human hippocampal CA_3_ and dentate gyrus. *Science*.

[10] Hopfield J. J. (1982). Neural networks and physical systems with emergent collective computational abilities. *Proceedings of the National Academy of Sciences of the United States of America*.

[11] Conklin J., Eliasmith C. (2005). A controlled attractor network model of path integration in the rat. *Journal of Computational Neuroscience*.

[12] Sompolinsky H., Crisanti A., Sommers H.-J. (1988). Chaos in random neural networks. *Physical Review Letters*.

[13] Wills T. J., Lever C., Cacucci F., Burgess N., O'Keefe J. (2005). Attractor dynamics in the hippocampal representation of the local environment. *Science*.

[14] Müezzinoglu M. K., Güzeliş C., Zurada J. M. (2005). An energy function-based design method for discrete Hopfield associative memory with attractive fixed points. *IEEE Transactions on Neural Networks*.

[15] Hurley M. (1998). Lyapunov functions and attractors in arbitrary metric spaces. *Proceedings of the American Mathematical Society*.

[16] Hélie S. (2008). Energy minimization in the nonlinear dynamic recurrent associative memory. *Neural Networks*.

[17] Koch C., Segev I. (2000). The role of single neurons in information processing. *Nature Neuroscience*.

[18] Robins A. V., McCallum S. J. R. (2004). A robust method for distinguishing between learned and spurious attractors. *Neural Networks*.

[19] Senn W., Fusi S. (2005). Learning only when necessary: better memories of correlated patterns in networks with bounded synapses. *Neural Computation*.

[20] Zemel R. S., Mozer M. C. (2001). Localist attractor networks. *Neural Computation*.

[21] Li G., Wen C., Zheng W. X., Chen Y. (2011). Identification of a class of nonlinear autoregressive models with exogenous inputs based on kernel machines. *IEEE Transactions on Signal Processing*.

